# Supplementary material for: Colossal negative magnetoresistance in field-induced Weyl semimetal of magnetic half-Heusler compound
Source: Nat Commun. 2023 Oct 10;14:6339. doi: 10.1038/s41467-023-41982-4 (PMC10564756; doi:10.1038/s41467-023-41982-4)
Supplement: Supplementary file 1 — Supplementary Information [file 41467_2023_41982_MOESM1_ESM.pdf]

Supplementary Information:

Colossal negative magnetoresistance in field-induced Weyl semimetal of magnetic half-Heusler compound

Kentaro Ueda,[1] Tonghua Yu,[1] Motoaki Hirayama,[1,2] Ryo Kurokawa,[1] Taro Nakajima,[2,3] Hiraku Saito,[3] Markus Kriener,[2] Manabu Hoshino,[2] Daisuke Hashizume,[2] Taka-hisa Arima,[2,4] Ryotaro Arita,[2,5] and Yoshinori Tokura[1, 2,6]

[1]*Department of Applied Physics and Quantum Phase Electronics Center (QPEC), University of Tokyo, Tokyo 113-8656, Japan*

[2]*RIKEN Center for Emergent Matter Science (CEMS), Wako 351-0198, Japan*

[3]*The Institute for Solid State Physics, University of Tokyo, Kashiwa 277-8561, Japan*

[4]*Department of Advanced Materials Science, University of Tokyo, Kashiwa 277-8561, Japan*

[5]*Research Center for Advanced Science and Technology, University of Tokyo, Komaba Meguro-ku, Tokyo 153-8904, Japan*

[6]*Tokyo College, University of Tokyo, Tokyo 113-8656, Japan*

I. Crystal characterization

Crystal shape and X-ray diffraction

HoAuSn single crystals were obtained by using Sn flux method as explained in Methods. R. Marazza et al. [1] employed the arc-melting method and reported that there are two structural phases of the lanthanide ternary compounds *R*AuSn; one is the hexagonal structure (see Fig. S2a) for *R*=La-Ho [2] and the other is the cubic type (Fig. 1a in the main text) for *R*=Ho-Lu. HoAuSn is located on the boundary between these phases. In fact, we obtain both structural types of crystals by changing the growth conditions. As we heated the ingredients in the vacuumed quarts up to 1150 ℃, cooled down at a rate of 3 ℃/h, and centrifuged them at 900 ℃, we obtained plate-like hexagonal shaped crystals (Fig. S1a). On the other hand, when we employed the low-temperature sequence as written in Methods, we obtained octahedron shaped crystals (Fig. S1b). Figures S1c and S1d show the x-ray diffraction patterns (XRD) with Cu Kα (λ=1.5418 Å) radiation for each crystal. We found that the hexagonal shaped crystals formed in the space group $P6_3mc$ (No. 186) while the octahedron shaped crystals formed in F-43m (No. 216) that was targeted in the present study. The lattice constant of cubic HoAuSn is 6.627 Å, which is between isostructural HoPtBi (~6.64 Å)[3] and HoPdBi (~6.60 Å)[4]. We used the Laue X-ray diffraction to check the orientation of samples as shown in Fig. S1e and S1f.

Physical properties of hexagonal HoAuSn

We can also distinguish these two structural phases by transport and magnetization properties. Figure S2b shows the temperature dependence of resistivity for hexagonal HoAuSn. With lowering temperature, the resistivity decreases down to less than 10 $\mu\Omega$cm with an anomaly at the magnetic transition temperature (~7 K). Such a metallic behavior at zero field is totally different from that of the half-Heusler type HoAuSn, as shown in Fig. 1b in the main text. Figure S2c shows the magnetic field dependence of magnetization at 2 K, which is well below the magnetic transition temperature. We observe a metamagnetic-like plateau in the intermediate magnetic-field range similar to the previous study on polycrystals [2], although the magnetization of our single crystal shows a sharper feature at the critical field.

Sample dependence of transport properties for cubic HoAuSn

Figure S3 shows the temperature dependence of resistivity for 5 samples. Similar trend is observed in all samples. At zero field, the resistivity gradually increases as the temperature increases. On the other hand, the resistivity decreases below 100 K as the external magnetic field is applied along the current direction. The residual resistivity at 14 T scatters from 10^{-5} to 10^{-7} Ωcm depending on samples, probably due more or less to lattice defects or impurities present in the respective samples.

II. Neutron scattering

Scan along the high-symmetry axes

Figure S4 shows neutron diffraction measurements along the high-symmetry axes (h,h,h), (h,h,0), and (0,0,l) at 1.65 K, which is well below T_N. We observed nuclear Bragg reflections at the reciprocal lattice points of (1,1,1), (2,2,0), and (0,0,2), which are allowed in the space group of HoAuSn (F-43m). These clear and sharp peaks indicate the high quality of the newly-synthesized HoAuSn single crystals. In addition to these peaks, we observe a magnetic peak at (0.5,0.5,0.5) below the magnetic transition temperature. As mentioned in Fig. 1e in the main text, the integrated intensity of the peak shows typical temperature dependence of an order parameter. These indicate that the spin propagation vector (q vector) is $\overrightarrow{q_1}$ =(0.5,0.5,0.5). We conclude that the magnetic moments are ferromagnetically aligned in (111) planes and stacked antiferromagnetically along the body diagonal, which is so called type-II antiferromagnetic structure on the face-centered cubic lattice, as reported in some other half-Heusler compounds with heavy R magnetic ions [5,6].

Owing to the cubic symmetry of the crystal, there are three other equivalent q vectors, specifically (0.5,0.5,−0.5), (0.5, −0.5,0.5), and (−0.5,0.5,0.5). In the magnetically ordered phase, there are four magnetic domains with respect to the q vectors, and two of them were observed on the (h,h,l) scattering plane in the present experiment.

Spin polarized neutron scattering analysis

To gain further insights into the spin orientation, we performed spin-polarized neutron scattering measurements. The neutron spin was polarized by using a Heusler polarizer perpendicular to the scattering plane. The polarized direction of the incident neutron is changed by the spin flipper set in front of the sample. The flipping ratio of R=14.42 was obtained by measuring the non-spin-flip (NSF) and spin-flip (SF) intensities of the direct beam. Figure S5a shows the SF intensity I_{SF} and NSF intensity I_{NSF} at the nuclear peak (1,1,1) scanning along (h,h,h). The effect of the imperfect beam polarization was corrected taking into account the above flipping ratio. We confirmed that I_{SF} is nearly zero, implying that the present spin polarization analysis works well.

Let us move on to the magnetic scattering. Here, $\vec{Q}$ is the neutron scattering vector, M_q is the Fourier-transformed spin component parallel to the spin propagation vector $\vec{q}$, M_z is that perpendicular to the scattering plane, and $M_\perp$ is that perpendicular to both z and $\vec{q}$ (see Fig. S5b). I_{NSF} and I_{SF} can be written as

$$I_{\mathrm{NSF}} \propto {M_z}^2,$$

$$\text{and } I_{\mathrm{SF}} \propto {M_q}^2\sin^2\alpha + {M_\perp}^2\cos^2\alpha,$$

where α is the angle between $\vec{Q}$ and $\vec{q}$ at the magnetic Bragg reflection. We measured three $\vec{Q}$; $\overrightarrow{Q_1}$=(0.5,0.5,0.5), $\overrightarrow{Q_2}$=(1.5,1.5,0.5), $\overrightarrow{Q_3}$=(0.5,0.5,1.5). The magnetic Bragg peaks at (1.5,1.5,0.5) and (0.5,0.5,1.5) correspond to the propagation vector $\overrightarrow{q_2}$=$(-0.5,\ -0.5,0.5)$, as shown in Fig. S4c. At $\overrightarrow{Q_1}$, where the angle α between $\overrightarrow{Q_1}$ and $\overrightarrow{q_1}$ is 0°, $I_{\rm SF}$ is proportional to $M_{\perp}{}^2$. On the other hand, $I_{\rm SF}$ is almost dominated by M_q at $\overrightarrow{Q_3}$, where $\alpha\sim 100°$ and thereby $\cos^2\alpha\sim 0.03$. As for $\overrightarrow{Q_2}$, $\theta\sim 132°$ and hence $I_{\rm SF}$ should involve both contributions almost equivalently.

Figures S5c-e show the rocking scan ($\Delta\omega$) profiles for $\overrightarrow{Q_1}$, $\overrightarrow{Q_2}$, and $\overrightarrow{Q_3}$, respectively. As mentioned in the main text, minimal $I_{\rm SF}$ is observed at $\overrightarrow{Q_3}$, indicating that the spin component along $\vec{q}$ (i.e. M_q) is negligible. On the other hand, $I_{\rm SF}$ is comparable to $I_{\rm NSF}$ at $\overrightarrow{Q_1}$, implying that the spin components perpendicular to $\vec{q}$ are isotropic. Taking into account the threefold rotational symmetry about the <111> axes in the cubic crystal structure, each q state should be composed of at least three domains regarding the orientation of the magnetic moments on the {111} plane.

III. Temperature dependence of Hall effect

Figure S6 shows the temperature dependence of Hall resistivity for low field (1 T) and high field (14 T), respectively. The Hall resistivity at 1 T significantly decreases and changes its sign with lowering temperature below 80 K, whereas those at 14 T sharply increases. It reflects that the magnetic field dependence of Hall resistivity shows a broad dip structure at low fields but shows the sign reversal at high fields below 80 K, as shown in Fig. 3b in the main text. This can be attributed to the drastic change of the Fermi surfaces induced by the exchange band splitting. As shown in the calculated band structures (Supplementary Fig. S11), the top of the heavy valence band (bottom of the conduction band) is above (below) the Fermi level at which the Ho moments are tilted by 10 degrees from AFM state. On the other hand, the top of the heavy valence band (bottom of the conduction band) shifts underneath (over) the Fermi level as the Ho moments are fully aligned, resulting in the disappearance of the hole and electron pockets. We note that the observed Hall effect may also include the anomalous Hall contribution stemming from the magnetic field-induced Weyl points, although they are very close to the pair in momentum space as shown in Fig. S9, and hence the anomalous Hall effect is quite small and possibly comparable to the normal Hall contribution.

IV. Crystalline orientation dependence

To check the relation between magnetotransport and crystalline orientations, we prepare another sample with a different geometry, as shown in the inset of Fig. S7a; the electric current flows along the [100] crystalline axis and the sample plane is (001). Figure S7a shows the temperature dependence

of resistivity at 0 T and 14 T (*H*//*I*). The resistivity at 14 T significantly decreases with lowering temperature and reaches less than 1 $\mu\Omega$cm, similar to the sample shown in Fig. 2 in the main text. Figures S7b, S7c, and S7d show the magnetic-field dependence of resistivity, Hall resistivity, and magnetization when the field is applied along the [001] axis. All of them show similar temperature and magnetic-field dependence to those shown in Figs. 3a-c. These indicate that the magnetotransport properties are isotropic with respect to the crystal orientation.

Figure S8 shows the magnetic field angle dependence of resistivity for two samples. One is the sample which is used in the main text (Fig. S8a) and the other is the sample shown in Fig. S7 (Fig. S8b). The electric current direction is [1-10] and the magnetic field is rotated from [1-10] to [111] in the former sample while the current direction is [100] and the magnetic field is rotated from [100] to [001] in the latter. The angle θ denotes the field direction against the current. Similar angle dependence is discerned in both samples. For instance, there is almost no angle dependence below 1.5 T. Above 1.5 T, the resistivity significantly decreases for $\theta = 0°$, whereas the resistivity shows clear upturn for $\theta = 90°$. These properties indicate that the magnetoresistance simply depends on the angle between the current and the field direction, rather than on the crystalline geometry.

V. Weyl points and large magnetoresistance

We have identified the presence of three Weyl points along the Γ-L direction in the FM state, with *M*//[111] (Fig S9a). Intriguingly, a total of six pairs of Weyl points exist in the vicinity of the Fermi level (−0.2 eV ~ 0.2 eV), forming two distinct groups positioned symmetrically with respect to the Γ point (Fig. S9b). The coordinates of symmetry-inequivalent nodes are (0.045 Å^{-1}, 0.045 Å^{-1}, 0.045 $^{-1}$), (0.039 Å^{-1}, 0.039Å^{-1}, 0.039 Å^{-1}), (0.035 Å^{-1}, 0.035 Å^{-1}, 0.035 Å^{-1}), and (0.047 Å^{-1}, 0.032 Å^{-1}, 0.035 Å^{-1}); equivalent positions can be obtained through inversion or C_3 symmetry. Notably, within each group, five nodes exhibit the same chirality, while one node displays the opposite chirality, as indicated by the color in Fig. S9b and corroborated by the Berry curvature distribution presented in Figs. S9c and S9d. Consequently, even in the scenario where the node with the minority chirality is annihilated, each group would still preserve a net chirality of ±4. This inherent robustness of adjacent Weyl points, sharing the same chirality, further amplifies the distinctive Weyl nature observed in the system. We note that such robustness of Weyl points is similar to the scenario in WP_2 (Ref. [12] of the main text).

One of the key consequences arising from Weyl points is spin-momentum locking. As an example, we examine the spin texture of the second and third highest valence bands (colored in blue in Fig. S10a) in proximity of E_F. The isosurfaces along with the associated spin texture are illustrated in Fig. S10b (with a magnified view in Fig. S10c). Additionally, we also provide a cross-sectional perspective (Fig. S10d, and the zoom-in view in Fig. S10e) to further enhance clarity. As centered around a Weyl

point, the spin undergoes a flip as momentum $\boldsymbol{k}$ is reversed (Fig. S10f), resulting in pronounced contrasting spin textures on the two surfaces. Within one surface, spin is also twisting as $\boldsymbol{k}$ varies, and spin-conversing scattering is prohibited for all channels except for $\boldsymbol{k}\rightarrow\text{-}\boldsymbol{k}$ (since the Weyl pair is enclosed in the same surface), the contribution of which to scattering is limited. As a result, electron scattering can be substantially reduced by virtue of the spin-momentum locking behavior, leading to high mobility and significant MR.

We show in Fig. S11 the evolution of band structure and Weyl points as the external magnetic field is applied. In Fig. S11b, slight tilting of magnetic moments (10º relative to the AFM configuration) can already induce Weyl point along the ΓZ direction, located in close proximity to the Γ point, with some of them lying above the Fermi energy E_F, although nearly invisible. This is the dawn of contribution from Weyl points to the negative magnetoresistance, as each pair aligns parallel to the field [7]. As the tilting angle is getting larger (corresponding to a stronger external field), all Weyl points with opposite chirality progressively separate and descend below E_F (Fig. S11c), giving rise to more linear-like dispersions and enhanced mobility of hole carriers, thus promoting the magnetoconductivity [8]. Eventually, the magnetic moments are aligned with the filed (i.e., FM state), as depicted in Fig. S11d, the pronounced Weyl nature leads to a significant negative magnetoresistance.

VI. Estimation of the relaxation time

The ab-initio band calculation reveals that there are four Fermi pockets (three holes and one electron) in the field-induced semimetal state. We observe several small peaks in the quantum oscillation (Fig. S12a). The Fermi wave vectors are 2.70×10^6/cm for the peak α_1, 5.24×10^6/cm for α_2, and 8.39×10^6/cm for β, which are reasonably close to the theoretical values (the former two correspond to the hole pockets and the last corresponds to the electron pocket). Figure S12b shows the temperature dependence of the oscillation amplitude for each peak. All of them are well fitted by the Lifshitz-Kosevich formula, with the masses of $0.33m_0$, $0.40m_0$, and $0.76m_0$, respectively. Only the largest hole Fermi surface is missing experimentally due perhaps to the higher required magnetic fields. Therefore, we use the theoretical value of 1.27×10^7/cm for k_F and the same mass of the other hole pocket α_2 ($0.40m_0$) for the effective mass. Using the residual resistivity of 0.18 $\mu\Omega$cm, we deduce the mean relaxation time $\tau_\mathrm{m} = 8.0 \times 10^{-11}$ s. Considering the strong intensity of α_2 in Shubnikov-de Haas oscillation, τ of α_2 pocket, which can be mostly affected by the Weyl points, is longer than τ_m.

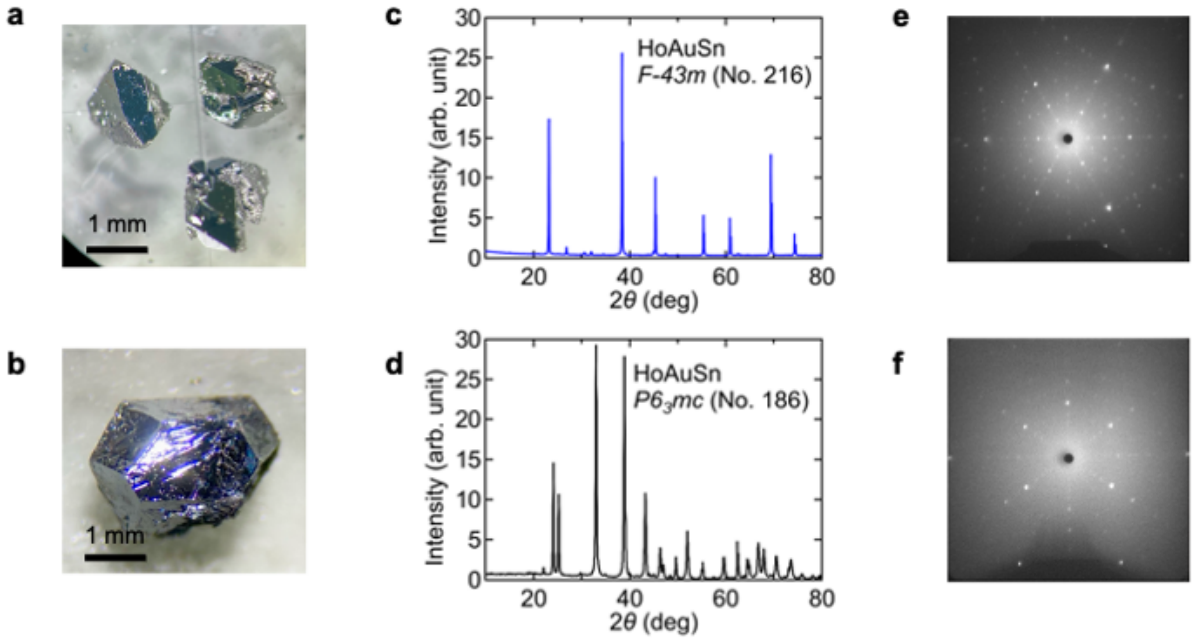

Figure S1. Sample characterization. a, Picture of cubic HoAuSn. **b,** Picture of hexagonal HoAuSn. **c,** powder X-ray diffraction pattern of cubic HoAuSn. **d,** powder X-ray diffraction pattern of hexagonal HoAuSn. **e**, Laue diffraction pattern of cubic HoAuSn for (111) plane. **f**, Laue diffraction pattern of cubic HoAuSn for (110) plane.

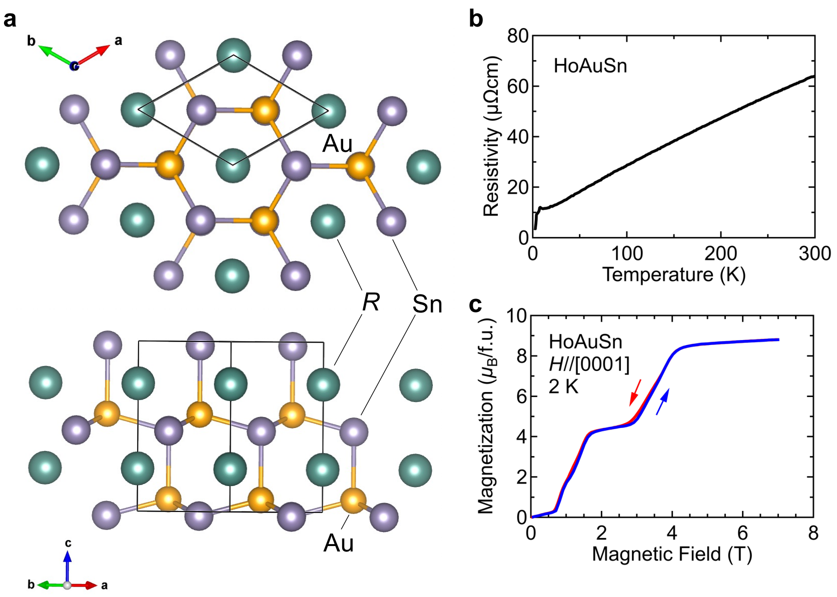

Figure S2. Physical properties of hexagonal HoAuSn. a, Crystal structure of hexagonal HoAuSn. The upper figure is the top view and the lower figure is the side view. The green balls denote Ho ion, the gold ones denote Au ion, and the silver ones denote Sn ion. **b,** Temperature dependence of resistivity for hexagonal HoAuSn. **c,** Magnetic field dependence of magnetization for hexagonal HoAuSn at 2 K.

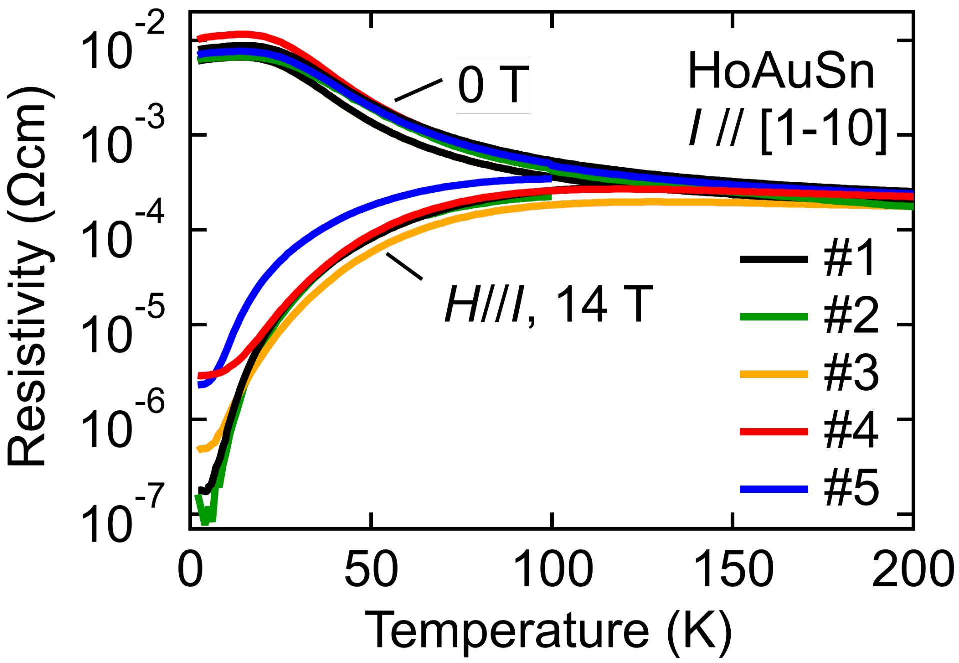

Figure S3. Temperature dependence of resistivity for several samples. The electric current *I* flows along [1-10] and the orthogonal plane is (111), same geometry as the sample in the main text. The sample #1 is used in the main text.

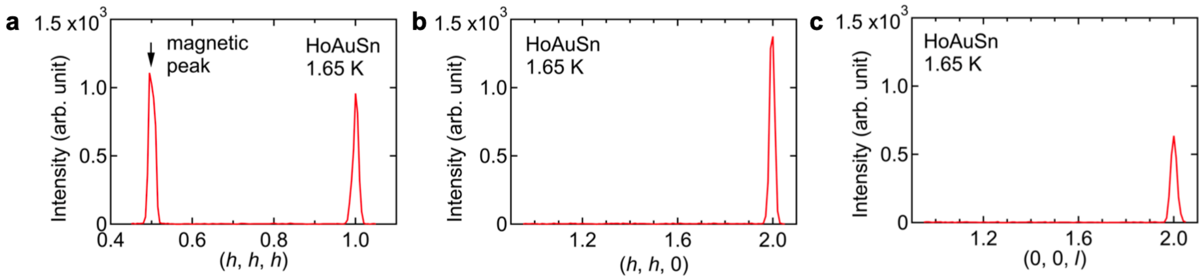

Figure S4. Neutron scattering profiles of HoAuSn scanning along high symmetry axes. **a,** Neutron scattering along [*h*,*h*,*h*] axis at 1.65 K. In addition to the nuclear scattering at (1,1,1), the magnetic peak (0.5,0.5,0.5) is observed. **b,** Neutron scattering along [*h*,*h*,0] axis at 1.65 K. **c,** Neutron scattering along [0,0,*l*] axis at 1.65 K.

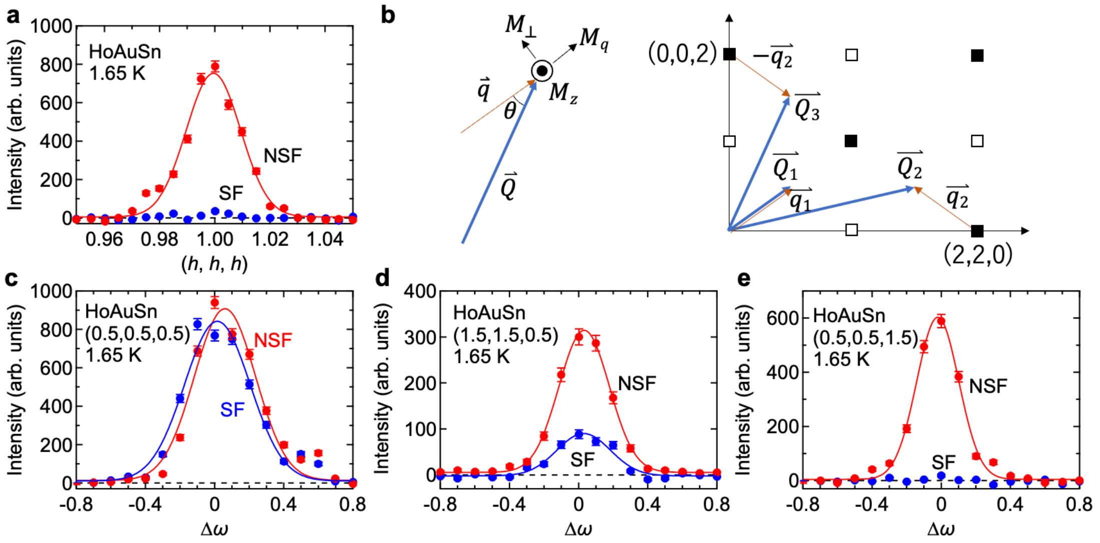

Figure S5. Neutron scattering with polarization analysis. SF stands for spin-flip scattering and NSF stands for non-spin-flip scattering. Solid lines are gaussian fitting curves. **a,** Neutron scattering around the nuclear peak (1,1,1) at 1.65 K. **b,** Geometry of neutron scattering. The scattering plane is (1,-1,0). $\vec{Q}$ is the neutron scattering vector, M_q is the spin component parallel to the spin propagation vector $\vec{q}$, $M_\perp$ is that perpendicular to $\vec{q}$, and M_z is that perpendicular to the scattering plane. Open and filled squares show the reciprocal lattice points where the nuclear reflections are forbidden and allowed, respectively. **c,** Rocking curve at (0.5,0.5,0.5) magnetic peak. **d,** Rocking curve at (1.5,1.5,0.5) magnetic peak. **e,** Rocking curve at (0.5,0.5,1.5) magnetic peak.

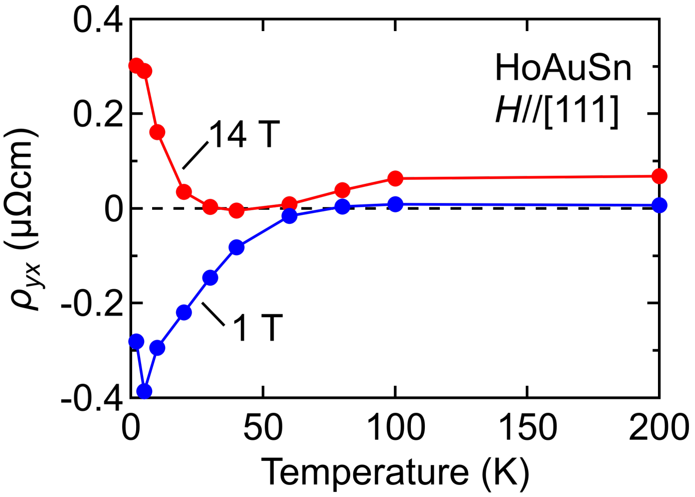

Figure S6. Temperature dependence of Hall resistivity. The blue marks are Hall resistivity at 1 T and the red marks are Hall resistivity at 14 T.

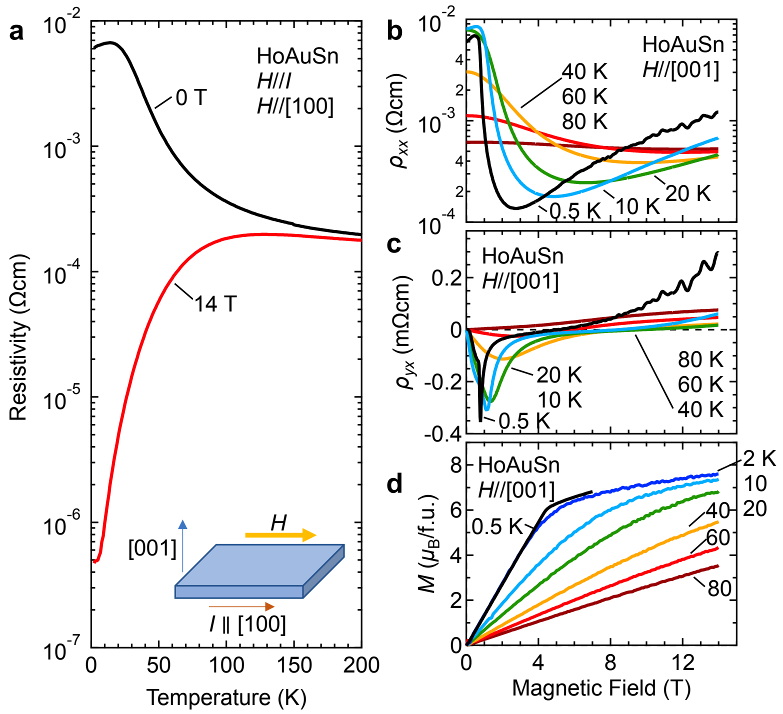

Figure S7. Transport properties of HoAuSn for different crystallographic orientation. The electric current I flows along [100] and the orthogonal plane is (001), as shown in the inset of **a**. **a,** Temperature dependence of resistivity at 0 T (black solid line), 14 T for H // I (red marks). **b,** Magnetic field dependence of resistivity for $H \perp I$ at several temperatures. **c,** Magnetic field dependence of Hall resistivity at several temperatures. **d,** Magnetic field dependence of magnetization at several temperatures.

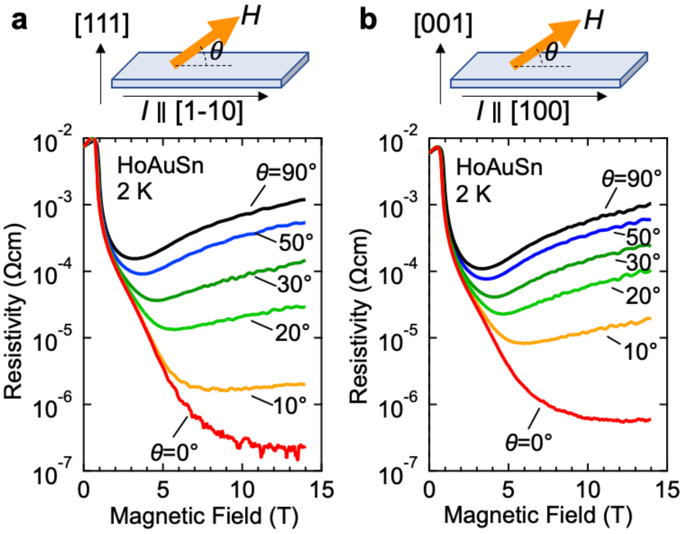

Figure S8. Magnetic field angle dependence of resistivity for two samples with different crystalline orientation. a, Magnetic field angle dependence of resistivity at 2 K for the sample which is used in the main text. The directions of the electric current and the magnetic field are shown in the drawing above the figure. **b,** Magnetic field angle dependence of resistivity at 2 K for the sample which is used in Fig. S6. The direction of the electric current and the magnetic field is shown in the drawing above the figure.

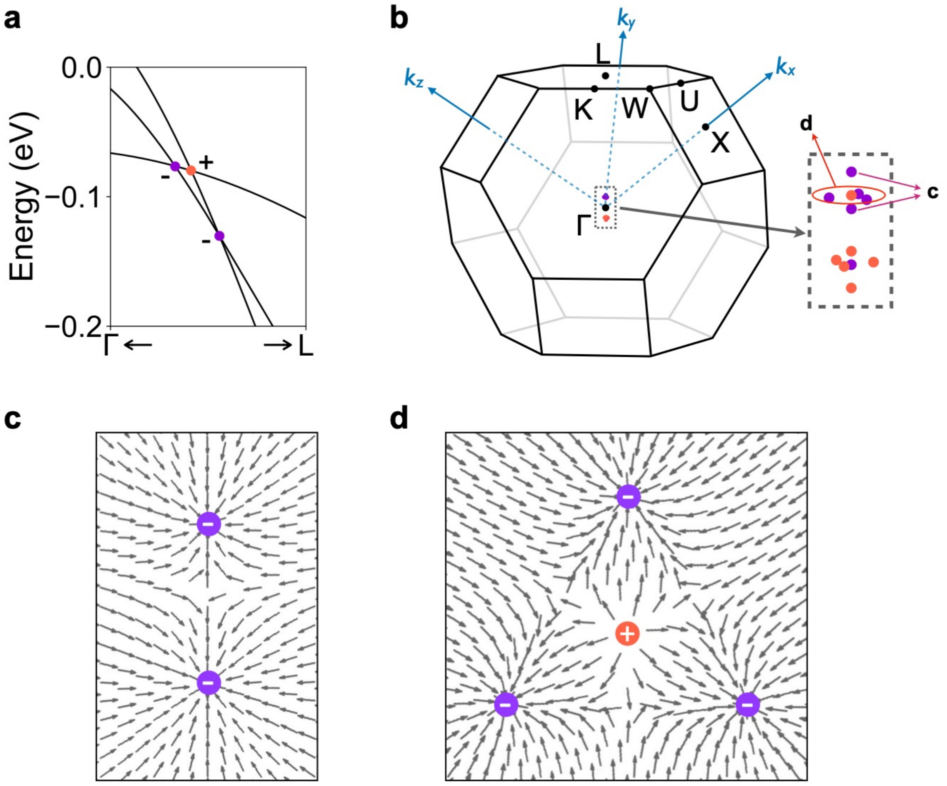

Figure S9. Weyl points and Berry curvature of HoAuSn in the FM state. a, The magnified view of the energy dispersed band structure along the Γ-L. The color denotes the sign of chirality for each degenerate point. **b,** The distribution of Weyl points in the first Brillouin zone. **c**, **d**, Distribution of Berry curvature on a plane parallel to the [111] direction (**c**) and that perpendicular to [111] (**d**). Weyl points are from the same group, with their position indicated in (**b**).

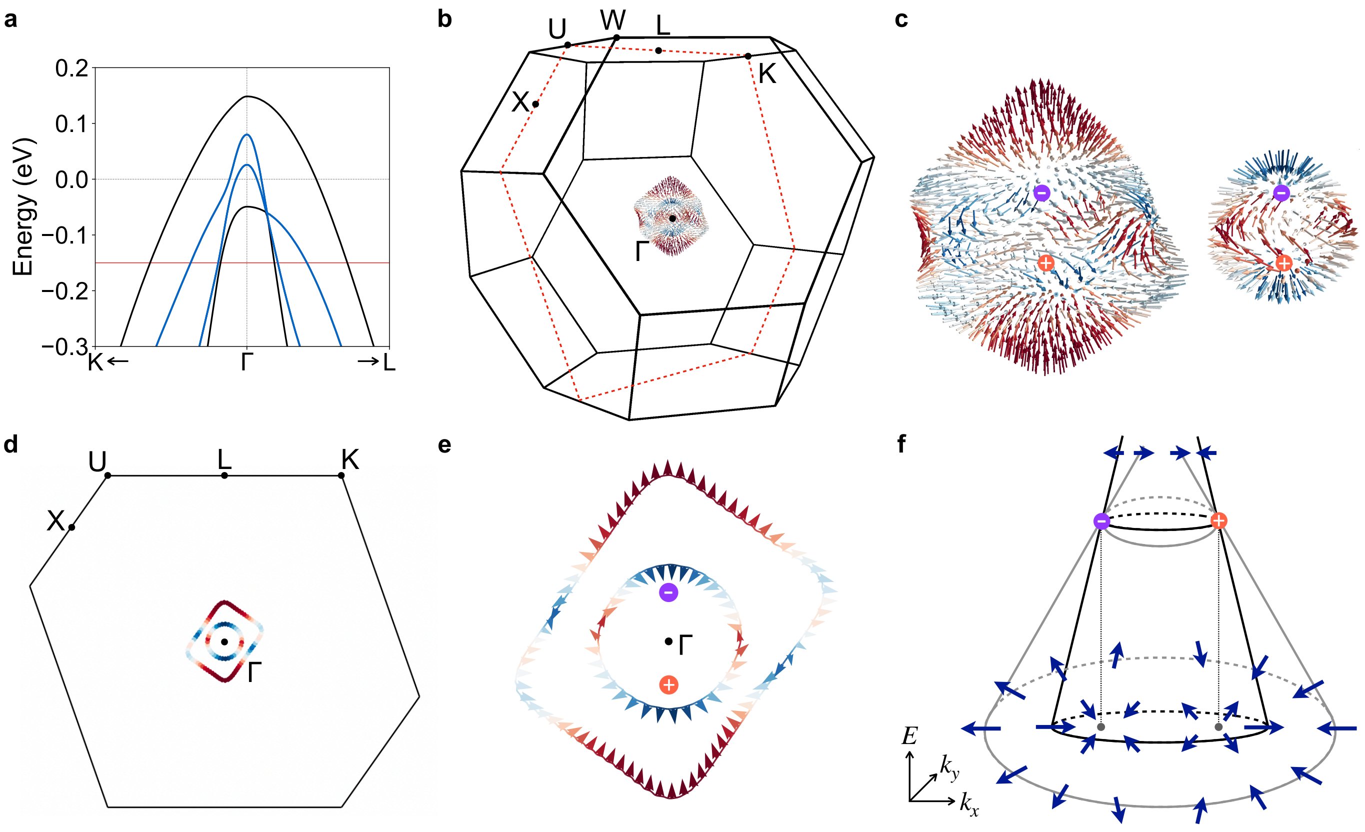

Figure S10. Spin texture of the FM HoAuSn. a, Band structure around the Γ point. **b,** Isoenergy surfaces of the second and third highest valence bands [highlighted in blue in (**a**)] and the associated spin texture. Red or blue represents spin parallel or antiparallel to [111], respectively. The energy level is -0.15 eV [*], marked in (**a**). **c,** Enlarged view of the isosurface of the second (left) and third (right) highest valence bands. Positions of Weyl points with respective chiralities are also indicated. **d,** Isoenergy slices and spin configuration on a Brillouin zone cross section [denoted in (**b**)]. **e,** Magnified view of the isoenergy slice. **f,** Schematic energy bands with a pair of Weyl points. Arrows stand for spins.

[*] Note that we choose the energy deviating from E_F, considering the fact that the isosurfaces at E_F are too small to show rich spin structures unless an ultra-dense ***k***-grid is used in the calculation; recall that the highest valence band having a fairly large Fermi surface manifests trivial spin texture. The spin texture at E_F is expected to bear a high resemblance with spin direction reversed, as implied in (**f**).

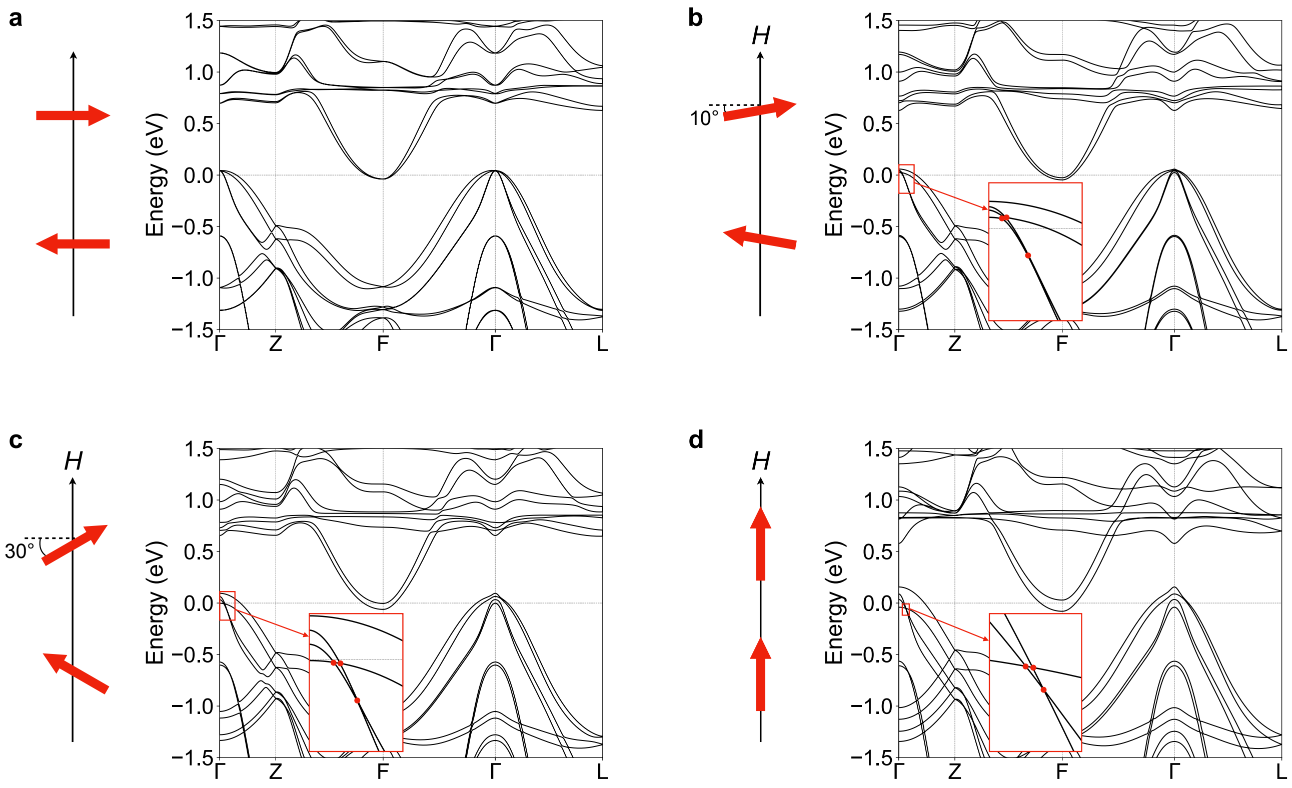

Figure S11. Evolution of energy bands and Weyl points as a function of magnetic field. The red thick arrows indicate the orientation of Ho moments which are controlled by the external magnetic field. The direction of the field is in parallel to Γ-Z direction. Red dots in the inset denote Weyl points.

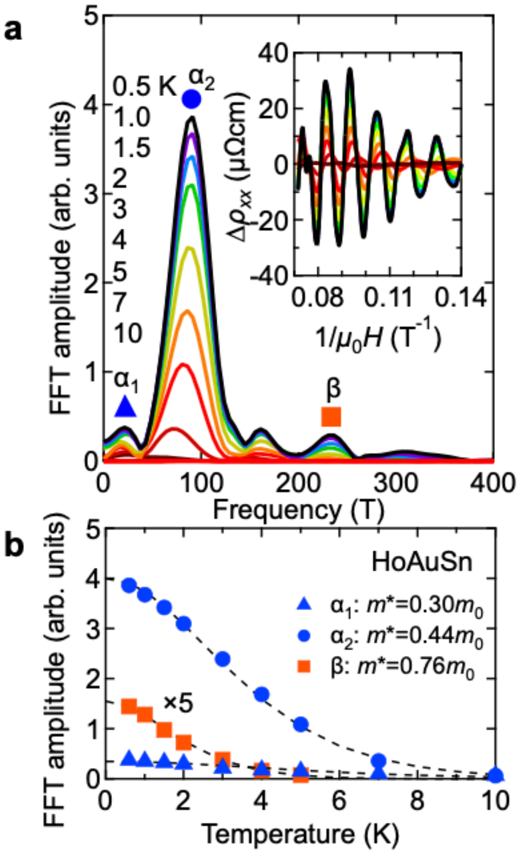

Figure S12. Quantum oscillations in HoAuSn. a, Fast-Fourier transformation amplitudes as a function of frequency. In the inset, the oscillatory part of the resistivity after subtracting the background (see text) is plotted as a function of $1/\mu_0 H$ for each temperature. The peak amplitudes are damped with increasing temperature (see panel d for the color code). **b,** Amplitude variation of the three peaks (marked with blue triangle, blue circle, and red square symbols, respectively) as a function of temperature. The dashed lines are fits to the data.

Reference

[1] Marazza, R., Rossi, D., and Ferro, R. On the ternary rare earth alloys: RAuPb compounds. *J. Less-Common Met.* **138**, 189-193 (1988).

[2] Baran, S., Leciejewicz, J., Ślaski, M., Hofmann, P., and Szytula, A. Magnetic properties of RAuSn compounds. *J. Alloys Compd.* **275**, 541-544 (1998).

[3] Canfield P. C. *et al.*, Magnetism and heavy fermion-like behavior in the *R*BiPt series. J. Appl. Phys. **70**, 5800 (1991).

[4] Nakajima, Y. *et al.*, Topological *R*PdBi half-Heusler semimetals: A new family of noncentrosymmetric magnetic superconductors. Sci. Adv. **1**, e1500242 (2015).

[5] Kreyssig, A., Kim, M. G., Kim, J. W., Pratt, D. K., Sauerbrei, S. M., March, S. D., Tesdall, G. R., Bund'ko, S. L., Canfield, P. C., McQueeney, R. J., and Goldman A. I. Magnetic order of GdBiPt studied by x-ray resonant magnetic scattering. Phys. Rev. B **84**, 220408(R) (2011).

[6] Müller, R. A., Lee-Hone, N. R., Lapointe, L., Ryan, D. H., Pereg-Barnea, T., Bianchi, A. D., Mozharivskyj, Y., and Flacau, R. Magnetic structure of GdBiPt: A candidate antiferromagnetic topological insulator. Phys. Rev. B **90**, 041109(R) (2014).

[7] Hirayama, M., Okugawa, R., & Murakami, S. Topological semimetals studied by ab initio calculations. J. Phys. Soc. Jpn. **87**, 041102 (2018).

[8] Shekhar, C. et al., Extremely large magnetoresisntance and ultrahigh mobility in the topological Weyl semimetal candidate NbP. Nat. Phys. **11**, 645 (2015).
